# Poster Session II - A269 INVESTIGATING USP18 DEFICIENCY IN PEDIATRIC INFLAMMATORY BOWEL DISEASE

**DOI:** 10.1093/jcag/gwaf042.269

**Published:** 2026-02-13

**Authors:** S Sorbara, N Warner, S Zhang, C Guo, J E O’Donnell, A Muise

**Affiliations:** Gastroenterology, Hepatology and Nutrition, The Hospital for Sick Children Department of Paediatrics, Toronto, ON, Canada; Gastroenterology, Hepatology and Nutrition, The Hospital for Sick Children Department of Paediatrics, Toronto, ON, Canada; Gastroenterology, Hepatology and Nutrition, The Hospital for Sick Children Department of Paediatrics, Toronto, ON, Canada; Gastroenterology, Hepatology and Nutrition, The Hospital for Sick Children Department of Paediatrics, Toronto, ON, Canada; Gastroenterology, Hepatology and Nutrition, The Hospital for Sick Children Department of Paediatrics, Toronto, ON, Canada; Gastroenterology, Hepatology and Nutrition, The Hospital for Sick Children Department of Paediatrics, Toronto, ON, Canada

## Abstract

**Background:**

Inflammatory bowel disease (IBD) is a lifelong, relapsing condition of the gastrointestinal tract. Pediatric-onset cases often involve stronger genetic and immune-mediated drivers than adult-onset disease. The Muise Lab focuses on identifying monogenic and rare variants underlying severe early-onset IBD, enabling precision diagnostics and therapies. One emerging candidate is *ubiquitin-specific peptidase 18* (USP18), a potent negative regulator of type I interferon (IFN-I) signaling. USP18 directly inhibits JAK1, limiting downstream STAT activation and preventing excessive inflammation. Deficiency or dysfunction could lead to chronic immune activation, ineffective viral control, and novel IBD subtypes. Our work centers on an index patient with markedly reduced USP18 expression and expands toward broader clinical and mechanistic characterization.

**Aims:**

(1) Define molecular and functional consequences of USP18 deficiency in pediatric IBD.

(2) Characterize IFN-I signaling and ISG dysregulation in patient-derived immune cells.

(3) Identify additional rare USP18 variants in pediatric IBD cohorts.

(4) Explore therapeutic approaches, including JAK inhibition, to restore immune balance.

(5) Build a scalable, genotype–phenotype database supporting precision medicine.

**Methods:**

Clinical data (CBC, CRP, calprotectin, imaging, immunophenotyping) are extracted from EPIC and compiled into a > 500-patient biobank for correlation with genomic findings. Cryopreserved PBMCs from the index patient were immortalized into EBV-derived lymphoblastoid cell lines, stimulated with IFN-α ± JAK inhibitor, and analyzed by Western blotting for USP18, pJAK1, and pSTAT1. RNA-seq demonstrating USP18 underexpression was validated by RT-qPCR across multiple ISGs. Whole-exome sequencing (WES) was reviewed to identify additional rare USP18 variants, with in silico pathogenicity predictions (CADD, Eugin) guiding downstream validation.

**Results:**

Preliminary qPCR shows decreased USP18 and ISG15 alongside increased RNASEL, consistent with disrupted negative feedback in IFN-I signaling. Lymphoblastoid lines were successfully generated, and ongoing immunoblotting will assess signaling dynamics and therapeutic response to JAK inhibition. WES has revealed a second pediatric IBD patient with a rare USP18 variant, currently under functional study. Clinical parameter extraction is complete for ∼20% of the biobank, creating infrastructure for broader cohort analyses.

**Conclusions:**

These findings support USP18 deficiency as a candidate driver of a genetically defined IBD subtype with aberrant IFN-I signaling. Integrating functional immunology, genomics, and clinical profiling, this work reflects the Muise Lab’s precision medicine strategy. If validated, therapeutic modulation of the JAK/STAT pathway or USP18 restoration could represent targeted interventions for USP18-related pediatric IBD.

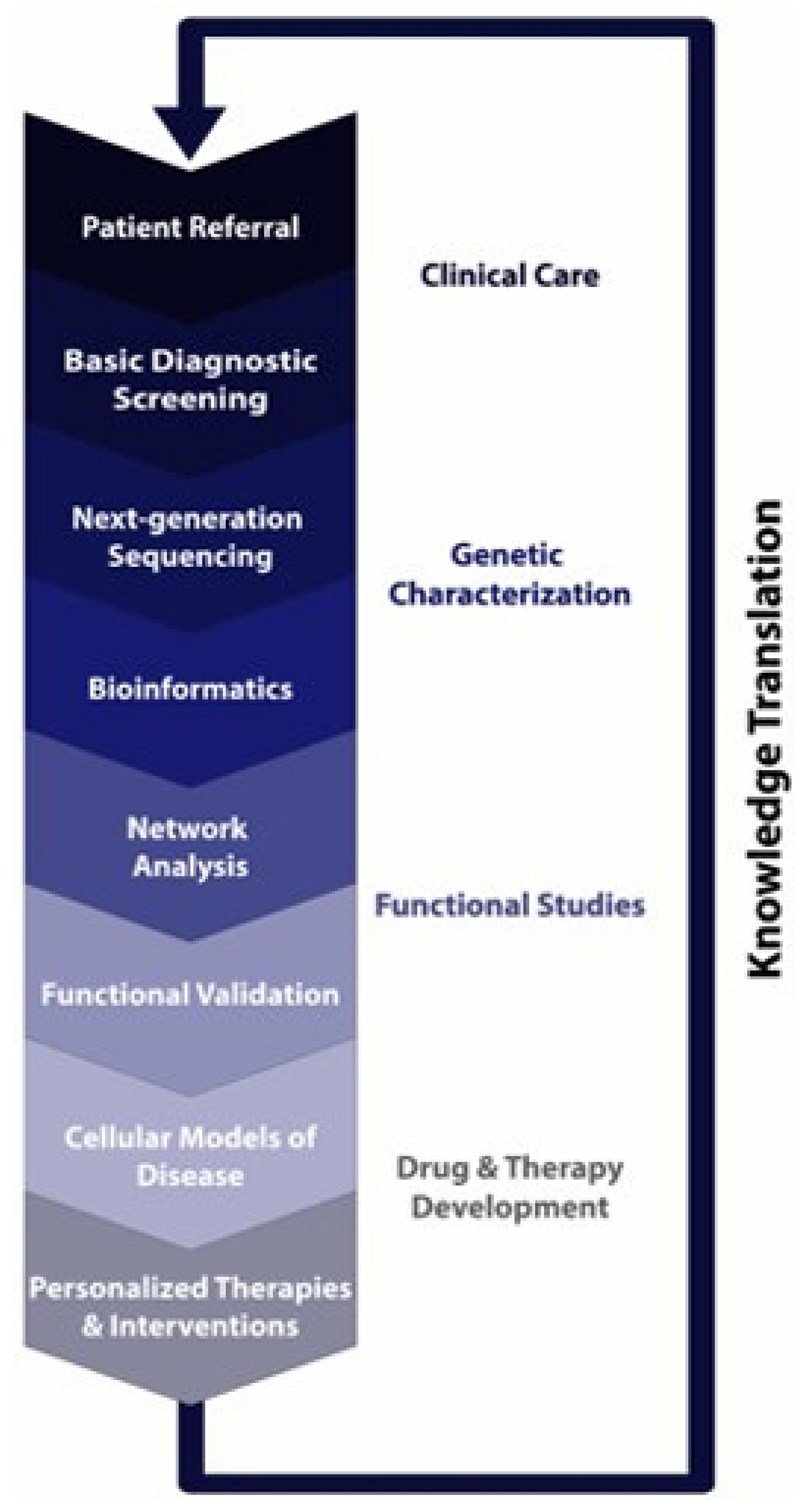

**Funding Agencies:**

CCC, CIHR, NRC

